# The sustainment of evidence-based adolescent substance abuse treatment in community settings

**DOI:** 10.1186/1940-0640-10-S1-A23

**Published:** 2015-02-20

**Authors:** Sarah B Hunter, Susan H Godley, Bryan R Garner, Bing Han, Lynsay Ayer, Mary Ellen Slaughter, Chau Pham

**Affiliations:** 1 Pardee RAND Graduate School, RAND Corporation, Santa Monica, CA, 90407, USA; 2 EBT Coordinating Center, Chestnut Health Systems, Normal, IL, 61761, USA; 3 Health Division, RAND Corporation, Arlington, VA, 22202-5050, USA; 4 Statistical Analysis, RAND Corporation, Pittsburgh, PA, 15213, USA

## Background

Numerous interventions for adolescent substance use disorders have been developed, tested, and supported by empirical evidence, yet of the 2 million 12- to 17-year-olds in need of treatment, only about 8 percent receive treatment. To help address this implementation gap, government agencies have offered time-limited discretionary grant funding to help facilitate delivery of evidence-based practices (EBPs). As one example, the Center for Substance Abuse Treatment (CSAT) has funded four cohorts of treatment organizations (16 in 2006; 17 in 2007; 15 in 2009; 34 in 2010) to deliver the Adolescent-Community Reinforcement Approach (A-CRA). However, evidence that such grants lead to longer-term EBP implementation (i.e., sustainment) beyond the initial grant period is limited. Additionally, there is lack of information regarding factors associated with EBP sustainment.

## Purpose

The current project is focused on addressing these gaps by: a) longitudinally characterizing A-CRA sustainment post-CSAT funding; and b) identifying factors (both prospectively and retrospectively) associated with A-CRA sustainment.

## Methods

During the first of three waves of data collection, which included both staff interviews and web surveys, information representing more than 80 percent of treatment programs was collected. Across the four cohorts, self-reported sustainment varied from 29 percent to 75 percent.

## Findings

The primary facilitators reported among programs that had sustained support longer than 1 year post-CSAT were executive-level support, successful funding from state or Medicaid sources, and modifications to the treatment, such as changing from an individual to group format. Noted barriers to sustainment included a lack of funding and staff turnover. The second wave of data collection is currently underway.

## Conclusions

The proposed research is important because a better understanding of the factors that influence EBP sustainability may lead to more effective dissemination strategies and ultimately improve the quality of care being delivered in community-based addiction treatment settings, leading to increased referral and receipt of treatment.

